# Aflatoxin Contamination in Agri‐Food Systems: A Comprehensive Review of Toxicity, Food Security, Economic Impacts, and Sustainable Mitigation Across the Value Chain

**DOI:** 10.1002/fsn3.71104

**Published:** 2025-10-16

**Authors:** Eyasu Yohannis, Markos Makiso Urugo, Tilahun A. Teka, Paulos Getachew, Yetenayet B. Tola, Sirawdink Fikreyesus Forsido, Yikeber Simachew Kebede, Tadesse Fikre Teferra

**Affiliations:** ^1^ Department of Food Science and Postharvest Technology College of Agricultural Sciences, Wachemo University Hossaena Ethiopia; ^2^ Department of Postharvest Management College of Agriculture and Veterinary Medicine, Jimma University Jimma Ethiopia; ^3^ Center for Food Science and Nutrition Addis Ababa University Addis Ababa Ethiopia; ^4^ School of Nutrition, Food Science, and Technology College of Agriculture, Hawassa University Hawassa Ethiopia; ^5^ Department of Food Science & Technology Texas A&M University College Station Texas USA

**Keywords:** aflatoxins, food systems, one health approach, toxicity

## Abstract

Aflatoxins (AFs) are highly toxic and carcinogenic secondary metabolites produced mainly by *Aspergillus flavus* and *Aspergillus parasiticus*, posing serious threats to global food safety, public health, and agricultural economies, particularly in tropical and subtropical regions. This review synthesizes current evidence on the types, sources, and mechanisms of AF toxicity, emphasizing their widespread prevalence in staple crops such as maize, groundnuts, and rice. Climate change, poor agricultural practices, and inadequate storage conditions exacerbate contamination risks. Chronic AF exposure is associated with hepatocellular carcinoma, immune suppression, stunted growth in children, and acute poisoning episodes, such as the 2004 Kenyan outbreak that resulted in 125 deaths. Economically, AFs lead to an estimated USD 6–18 billion in annual losses due to trade rejections, healthcare costs, and productivity decline. This review also explores recent advances in sustainable and innovative mitigation strategies across pre‐ and post‐harvest stages. These include the use of atoxigenic biocontrol strains, genetic resistance, natural inhibitors, hermetic storage technologies, nanotechnology, biological detoxification, and climate‐smart agricultural practices. Additionally, emerging digital tools such as Internet of Things (IoT) sensors, blockchain‐based traceability, and machine learning models offer promising approaches for real‐time monitoring and predictive risk assessment. Despite these advancements, major challenges remain, including limited detection infrastructure, low awareness among smallholder farmers, and fragmented policy enforcement. The review concludes by emphasizing the need for integrated, multi‐sectoral interventions anchored in a One Health approach to reduce aflatoxin risks, promote food security, and safeguard human and environmental health globally.

## Graphical abstract

Toxic and carcinogenic aflatoxins from Aspergillus contaminate global staple crops, threatening food security, public health, and causing $6–18B in annual economic losses. Climate change, poor agricultural and post‐harvest practices, and socioeconomic constraints intensify aflatoxin contamination risks. Sustainable mitigation strategies from biocontrol and genetic resistance to advanced post‐harvest and digital tech offer significant potential to reduce aflatoxin contamination.

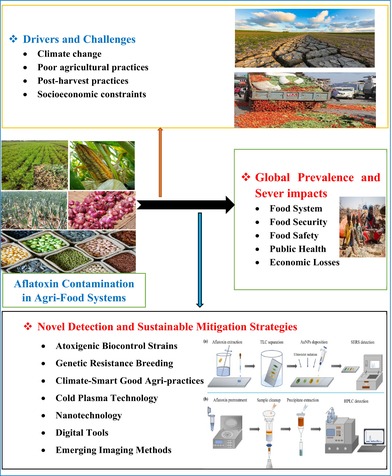

## 1 Introduction

Agri‐food systems are inherently complex, multidimensional, and cross‐sectoral. Understanding their dynamic interactions and evaluating their performance is essential to enhancing their contribution to the Sustainable Development Goals (SDGs) (Adamu Demelash and Abate Alemu 2024; FAO 2018; Zhu et al. 2018). These systems are shaped by a range of interconnected factors, including production, consumption, trade, and distribution (Adamu Demelash and Abate Alemu 2024). Ensuring food quality, nutrition, safety, and security for current and future generations requires the establishment of sustainable food systems that integrate environmental, economic, social, and health considerations (FAO 2018; Fung et al. 2018; Zhu et al. 2018). Achieving this vision necessitates transformative changes in how food is produced, processed, distributed, and consumed, as well as the development and alignment of policies and strategies that support resilient and equitable food systems.

One of the critical challenges to achieving food safety and security is microbial contamination, particularly by mycotoxins. The modern food supply chain is intricate, involving numerous actors and stages from production and processing to storage and distribution, each presenting potential points of contamination (Bou‐Mitri et al. 2018; Zhu et al. 2018). In developing regions, especially rural areas, mycotoxin contamination poses a significant threat, exacerbating existing food quality and safety concerns. Mycotoxins are toxic secondary metabolites produced by molds such as *Aspergillus, Fusarium, Penicillium*, and *Alternaria*, and are characterized by low molecular weight (300–700 Da) (Balan et al. 2024; Iqbal 2021; Thanushree et al. 2019). Contamination can occur at any stage from field to fork, leading to significant food waste and imposing social and economic burdens on food systems (Balan et al. 2024; Liu et al. 2022).

Among the over 400 identified mycotoxins, aflatoxins (AFs) are among the most potent and widespread, particularly due to their toxicity and economic impact. Produced predominantly by *Aspergillus flavus* and *A. parasiticus*, AFs are immunosuppressive, mutagenic, teratogenic, and highly carcinogenic (Balan et al. 2024; Nazareth et al. 2024; Romero‐Sánchez et al. 2024). They frequently contaminate staple crops such as maize, groundnuts, rice, and spices, and can also accumulate in animal‐derived products like milk, meat, and eggs through contaminated feed (Mengesha et al. 2024; Pleadin et al. 2021; Tadesse et al. 2020). AFs are categorized into several subtypes, with B1, B2, G1, G2 (from crop contamination) and M1, M2 (from animal products) being of particular concern due to their fluorescence under UV light and their relevance to food safety (Liu et al. 2022; Jallow et al. 2021; Omara et al. 2020; Singh and Mehta 2020).

Aflatoxin B1 (AFB_1_) is the most prevalent and toxic form, with a strong genotoxic and carcinogenic profile (Balan et al. 2024; Jallow et al. 2021). Environmental conditions such as high temperature, humidity, poor storage, and inadequate post‐harvest handling exacerbate aflatoxin synthesis and cross‐contamination (Romero‐Sánchez et al. 2024; Jallow et al. 2021; Omara et al. 2020). The risk is further magnified by climate change, which alters fungal ecology and expands the geographic distribution of toxigenic species (Casu et al. 2024; Loi et al. 2023; Perera 2018). Exposure to AFs through contaminated food and feed is associated with severe health effects, including hepatotoxicity, immunotoxicity, and cancer, particularly hepatocellular carcinoma (HCC) (Balan et al. 2024; Kumar et al. 2021; Mahato et al. 2019). Although AFM1 is considered less toxic than AFB_1_, it still presents considerable health risks (Balan et al. 2024). Regulatory limits for AF levels in food and feed have been established by various countries and international bodies. For instance, the European Union (EU) enforces strict thresholds of 2 μg/kg for AFB_1_ and 4 μg/kg for total AFs, while the U.S. and World Food Programme (WFP) maintain limits of 20 and 10 μg/kg, respectively (Balan et al. 2024; Meneely et al. 2023; Mahato et al. 2019; EU 2010).

Numerous pre‐harvest and post‐harvest strategies have been developed to control AF contamination. Pre‐harvest approaches, including good agricultural practices (GAPs), biological control agents, and the development of resistant crop varieties, aim to reduce fungal growth in the field (Balan et al. 2024; Liu et al. 2022). Post‐harvest methods such as proper drying, sorting, cleaning, and storage are critical to limiting further contamination (Leslie et al. 2021). However, the thermal stability of AFs at high temperatures (237°C–306°C) limits the effectiveness of many traditional processing technologies (Pankaj et al. 2018). Consequently, there has been a growing interest in novel and sustainable detoxification methods, including biological, chemical, and physical interventions, as well as biotechnological and genetic approaches (Jallow et al. 2021; Kumar et al. 2021; Pankaj et al. 2018). This review aims to provide a comprehensive synthesis of recent scientific developments related to AF contamination in global agri‐food systems. It explores the nature, occurrence, mechanisms of toxicity, and the health, food security, and economic impacts of AFs. Furthermore, it emphasizes innovative and sustainable mitigation strategies across the value chain, highlighting emerging technologies and integrated approaches to AF prevention and control.

## 2 Sources, Types and Chemistry of AFs

AF contamination in agricultural commodities results from a complex biochemical process that begins with the invasion of crops by aflatoxigenic fungi, primarily Aspergillus species, followed by the biosynthesis of toxins in the infected tissues (Okechukwu et al. 2024; Jallow et al. 2021; Gizachew et al. 2019; Mahato et al. 2019). The initiation and progression of fungal colonization and AF production are governed by several factors, including environmental conditions, crop species, and ecological dynamics (Negash 2018). Consequently, the extent and severity of AF contamination are largely shaped by the fungal ecology within the crop production environment. The Aspergillus genus is genetically diverse, comprising species with varying AF‐producing capacities (Kjærbølling et al. 2020; Taniwaki et al. 2018).

Current literature identifies approximately 24 aflatoxigenic species within the Aspergillus genus, predominantly grouped into three taxonomic sections: *Flavi, Nidulantes*, and *Ochraceorosei* (Urugo and Woldegiorgis 2022; Bartholomew et al. 2021). Among these, *A. flavus* and *Aspergillus parasiticus* are the principal AF producers, significantly affecting food safety and public health worldwide (Alemu Degefe and Geleta 2024; Seval and Hakan 2022; Kumar et al. 2021). Cereals such as maize, rice, sorghum, and millet are particularly prone to AF contamination, especially under warm and humid climatic conditions (Alameri et al. 2023). For example, AFs have been detected in 92.9% of millet, 67.9% of maize, and 50% of sorghum samples stored under poor post‐harvest conditions (Balan et al. 2024; Shabeer et al. 2022; Bartholomew et al. 2021). Oilseeds, including peanuts and soybeans, are similarly vulnerable, often exceeding regulatory safety thresholds (Meneely et al. 2023; Mahato et al. 2019). These findings underscore the critical role of post‐harvest handling, storage practices, and environmental factors in AF prevalence (Bhardwaj et al. 2023; Umar et al. 2023).

AFs regulatory safety thresholds differ widely across countries and depend on the type of food or product, but generally range from 0.5 to 20 parts per billion (ppb) for total AFs in food (Kinyenje et al. 2023; Benkerroum 2020a; Ali 2019). For instance, the EU enforces some of the strictest limits, setting maximum levels for total AFs (TAFs) in food at 4 μg/kg and for aflatoxin B_1_ at 2 μg/kg, especially in products intended for direct human consumption. In contrast, the United States Food and Drug Administration (FDA) permits higher limits, with 20 μg/kg for TAFs in most food products and 0.5 μg/kg in milk. The East African Community follows a similar standard to the U.S., with 20 μg/kg as the maximum for many foodstuffs.

AF contamination can persist beyond the primary production stage, extending into animal‐derived foods. Livestock fed with contaminated feed can bio‐accumulate AFs in tissues, particularly in the liver, and excrete metabolites like AFM1 in milk (Alamir et al. 2023; Morshdy et al. 2023; Pleadin et al. 2021; Tadesse et al. 2020; Mahato et al. 2019). In some cases, fungal growth may occur directly on meat products or via contaminated processing additives (Aljazzar et al. 2021). This pathway highlights the importance of comprehensive monitoring and control strategies that encompass not only crops but also animal feed and products to safeguard food systems (Mengesha et al. 2024). To effectively mitigate AF‐related risks, it is essential to have a holistic understanding of the sources and mechanisms of AF contamination across the food value chain.

### 2.1 Types and Chemical Characteristics of AFs

AFs are a group of structurally related secondary metabolites produced predominantly by *A. flavus* and *Aspergillus parasiticus*. These compounds pose significant health risks to humans and animals and are associated with substantial economic losses globally (Seval and Hakan 2022; Pickova et al. 2021; Marchese et al. 2018). The major naturally occurring AFs include B1 (AFB1), B2 (AFB2), G1 (AFG1), G2 (AFG2), and their hydroxylated derivatives M1 (AFM1) and M2 (AFM2) (Alameri et al. 2023; Elkenany and Awad 2021). *A. flavus* predominantly produces AFB1 and AFB2, while *A. parasiticus* synthesizes both B‐ and G‐types (Khabbouchi et al. 2024). AFM1 and AFM2 are formed in the liver of animals following the ingestion of AFB1 and AFB2, respectively, and are subsequently excreted in milk and dairy products (Nazareth et al. 2024; Shabeer et al. 2022). These compounds exhibit distinct fluorescence properties under ultraviolet (UV) light, aiding in their detection and differentiation.

Among these, AFB1 is the most potent and toxic, classified as a Group 1 carcinogen by the International Agency for Research on Cancer (IARC) due to its strong genotoxic and hepatocarcinogenic effects (Pozarska et al. 2024; Martínez et al. 2023). It is commonly found in a variety of food commodities, including cereals, oilseeds, spices, and nuts (Alameri et al. 2023; Kumar et al. 2021). AFG1, although less potent than AFB1, remains a significant health concern, while AFM1 is particularly hazardous for infants and young children due to its thermal stability and persistence in milk products (Al Tamim et al. 2024; Alameri et al. 2023; Tadesse et al. 2020).

### 2.2 Chemistry and Physicochemical Properties of AFs

Chemically, AFs share a difuranocoumarin backbone, with structural variations accounting for differences in toxicity, fluorescence, and solubility (Marchese et al. 2018). B‐type AFs (AFB1 and AFB2) contain a cyclopentanone ring, while G‐types (AFG1 and AFG2) possess a six‐membered lactone ring. These differences lead to distinct fluorescent characteristics under UV light—blue for B‐types and green for G‐types. The subscript numbers (1 and 2) indicate the relative abundance and toxicity, with “1” denoting the more prevalent and potent form (Wang et al. 2022; Ghalkhani et al. 2019; Sharma et al. 2018).

**FIGURE 1:**
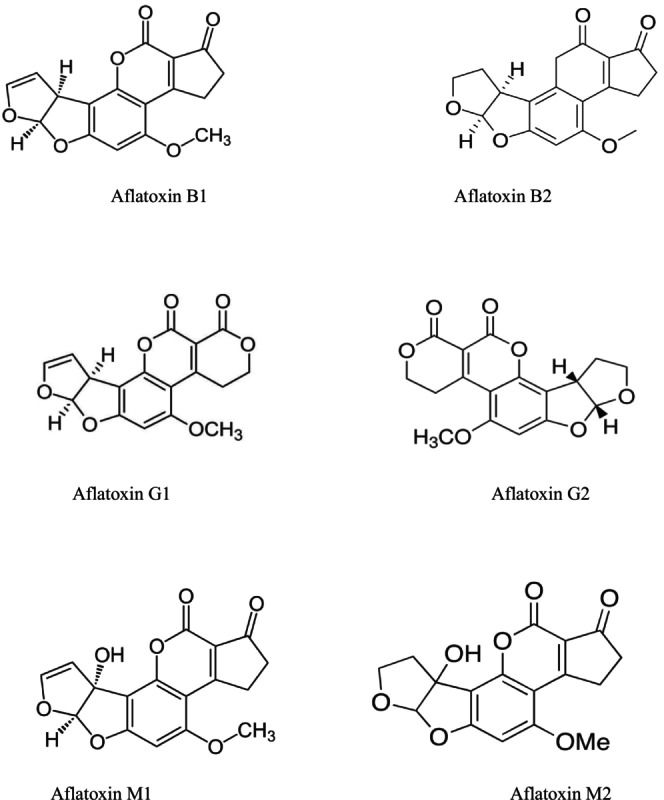
Chemical structure of major types of aflatoxins (Jallow et al. 2021; Mahmoud et al. 2025; Okechukwu et al. 2024; Popescu et al. 2022).

AFs are typically colorless to pale yellow crystalline compounds. They fluoresce under UV light—blue (AFB1, AFB2), green (AFG1, AFG2), and blue‐violet (AFM1) (Marchese et al. 2018). They exhibit limited water solubility (10–20 μg/mL) but are highly soluble in moderately polar solvents such as chloroform, methanol, and dimethyl sulfoxide. Chemically, AFs are unstable under UV light in the presence of oxygen and are sensitive to extreme pH conditions (< 3 or > 10), with the lactone ring opening under alkaline conditions—a reaction reversible by acidification (Ghalkhani et al. 2019). Thermal stability represents a major challenge in AF detoxification. AFs withstand high processing temperatures (> 100°C), rendering conventional heat treatments like pasteurization largely ineffective, particularly for products such as milk and dairy (Djekic et al. 2020). Alkaline ammoniation can irreversibly detoxify AFs by opening the lactone ring and causing decarboxylation, yet this method requires strict regulation due to possible nutritional and safety concerns. Key physicochemical and toxicological properties of major AFs are summarized in Table 1. The structural representations of AFB1, AFB2, AFG1, AFG2, AFM1, and AFM2 are shown in Figure 1.

**TABLE 1:** A summary of the physical and chemical properties and toxicological profiles of major types of aflatoxins.

Aflatoxins	Molecular formula	Molecular weight (g/mol)	Melting point (°C)	Toxicity levels: LD_50_ (mg/kg)	Test organism/exposure route	Descriptions and adverse effects	References
Aflatoxin B_1_	C_17_H_12_O_6_	312.3	268–269	0.24–60	Chick embryo (injection); various animals (oral)	The most potent and prevalent aflatoxin; genotoxicity, carcinogenicity (IARC Group 1), hepatotoxicity, teratogenicity, immunotoxicity, DNA damage	Al Tamim et al. (2024), Popescu et al. (2022), Jallow et al. (2021), Benkerroum (2020a), and Cho et al. (2020)
Aflatoxin B_2_	C_17_H_14_O_6_	314.3	286–289	84.8	Duckling (oral)	It is probably less toxic than B1 but still dangerous; carcinogenicity, hepatotoxicity, weak mutagenicity	Al Tamim et al. (2024), Balan et al. (2024), Platt‐samoraj (2022), Jallow et al. (2021), and Benkerroum (2020a)
Aflatoxin G_1_	C_17_H_12_O_7_	328.3	244–246	39.2	Duckling (oral)	Marked by their green fluorescence under UV light, whereas B_1_ fluoresce blue; hepatotoxicity, carcinogenicity (IARC Group 1), DNA adduct formation	Al Tamim et al. (2024), Balan et al. (2024), Shabeer et al. (2022), Jallow et al. (2021), and Benkerroum (2020a)
Aflatoxin G_2_	C_17_H_14_O_7_	330.3	237–240	172.5	Duckling (oral)	Marked by their green fluorescence under UV light, whereas B_2_ fluoresce blue; low toxicity, weak mutagenicity, hepatotoxicity (lower potency than G_1_)	Al Tamim et al. (2024), Balan et al. (2024), Shabeer et al. (2022), Jallow et al. (2021), and Benkerroum (2020a)
Aflatoxin M_1_	C_17_H_12_O_7_	328.3	297–299	16.6	Mouse (oral), duckling (oral)	A hydroxylated metabolite of aflatoxin B_1_; hepatotoxicity, nephrotoxicity, carcinogenicity (IARC Group 2B), immunosuppression; found in milk and dairy products from animals consuming contaminated feed	Balan et al. (2024), Jallow et al. (2021), Platt‐samoraj (2022), Shabeer et al. (2022), and Benkerroum (2020a)
Aflatoxin M_2_	C_17_H_14_O_7_	330.3	293	62	Duckling (oral)	A hydroxylated metabolite of aflatoxin B2, less toxic and prevalent than AFM1; low toxicity, weak carcinogenicity, low hepatotoxicity, potential carcinogen (limited evidence), less studied	Balan et al. (2024), Shabeer et al. (2022), Jallow et al. (2021), and Shehab et al. (2019)

*Note:* The International Agency for Research on Cancer (IARC) classifies agents based on their potential to cause cancer, using four groups: Group 1 (carcinogenic to humans), Group 2A (probably carcinogenic to humans), Group 2B (possibly carcinogenic to humans), and Group 3 (not classifiable as to carcinogenicity to humans).

## 3 Toxicity and Mechanisms of Action of AFs

AFs are among the most extensively studied mycotoxins due to their potent toxicity and diverse adverse health effects. These include acute toxicities such as immunosuppression, allergic reactions, and hepatitis, as well as chronic impacts such as hormonal imbalances, infertility, nephrotoxicity, teratogenicity, and carcinogenicity (Romero‐Sánchez et al. 2024; El‐Sayed et al. 2022). AFB1, in particular, poses a significant threat to global public health owing to its widespread occurrence in food and its potent carcinogenic potential (Rushing and Selim 2019). The IARC has classified AFB1 as a Group 1 human carcinogen, with a toxicity hierarchy of B1 ≫ G1 > B2 > G2. Chronic exposure to AFB1, especially through contaminated diets, has been strongly associated with the development of HCC, particularly in regions lacking effective food storage systems and regulatory frameworks (Moloi et al. 2024; Romero‐Sánchez et al. 2024; El‐Sayed et al. 2022).

The toxicological effects of AFB1 are primarily mediated through its metabolic activation in the liver. Cytochrome P450 enzymes, notably CYP3A4 and CYP1A2, convert AFB1 into a highly reactive intermediate, AFB1‐8,9‐epoxide (Moloi et al. 2024; Yilmaz and Bag 2022). This epoxide binds covalently to DNA, forming mutagenic adducts, particularly at codon 249 of the TP53 tumor suppressor gene—a mutation frequently observed in HCC cases linked to AF exposure. In addition to its genotoxicity, AFB1 contributes to liver damage through oxidative stress, generating reactive oxygen species (ROS) that damage cellular lipids, proteins, and nucleic acids (Moloi et al. 2024; Marchese et al. 2018). This oxidative burden disrupts cellular function and triggers apoptosis. Furthermore, AFB1 impairs mitochondrial activity, resulting in diminished ATP synthesis and activation of intrinsic apoptotic signaling pathways (Moloi et al. 2024; Yilmaz and Bag 2022). These mechanisms synergistically exacerbate liver injury and promote tumor development.

AFB1 also exerts immunotoxic effects, impairing both innate and adaptive immune responses. Studies have documented reduced lymphocyte proliferation, dysregulated cytokine expression, and heightened vulnerability to infections in individuals exposed to AFs (Dai et al. 2024; Khan et al. 2021; Benkerroum 2020a, 2020b). Additionally, emerging evidence indicates that AFB1 may induce neurotoxicity. It is capable of crossing the blood–brain barrier, where it induces oxidative stress and neuronal apoptosis, leading to behavioral and cognitive impairments, as demonstrated in animal models (Dai et al. 2024; Moloi et al. 2024; El‐Sayed et al. 2022; Marchese et al. 2018).

Epidemiological research, particularly in sub‐Saharan Africa (SSA), has linked chronic AF exposure to growth retardation and protein‐energy malnutrition syndromes such as kwashiorkor. These outcomes are likely due to AF‐induced intestinal damage and reduced nutrient absorption (Brevik et al. 2024; Rasheed et al. 2021; Misihairabgwi et al. 2019). Although acute aflatoxicosis is relatively rare, it can be fatal. A notable case occurred during a 2004 outbreak in Kenya, where consumption of highly contaminated maize resulted in over 125 deaths due to acute hepatitis and jaundice (Jacob et al. 2022; Benkerroum 2020a; Misihairabgwi et al. 2019).

Humans are primarily exposed to AFs through ingestion, although inhalation and dermal absorption are also possible. Oral exposure remains the most significant and harmful route due to the widespread contamination of staple foods and the limited efficacy of industrial processing methods in completely eliminating AFs (Romero‐Sánchez et al. 2024). A detailed overview of the different types of AFs, their primary food contaminants, associated toxic effects, and mechanisms of action is presented in Table 2, offering a comprehensive reference for understanding their multifaceted impact on human health. Given the ubiquity and toxicity of AFs, there is an urgent need for integrated mitigation strategies. These include the adoption of GAPs to prevent fungal growth, the establishment of robust food safety regulations, and the development of effective detoxification technologies (Dai et al. 2022; Li et al. 2022; Benkerroum 2020b; Rushing and Selim 2019). A multi‐pronged approach involving public health interventions, regulatory enforcement, and stakeholder engagement is essential to reduce AF‐related health risks and ensure food safety across vulnerable populations.

**TABLE 2:** Overview of the different types of aflatoxins, their primary food contaminants, associated toxic effects, and mechanisms of action.

Aflatoxin type	Main producers	Primarily contaminated foods	Toxicity and associated diseases	Mechanism of action	References
Aflatoxin B1 (AFB1)	*Aspergillus flavus*; *Aspergillus parasiticus*; *Aspergillus nomius*	Maize, peanuts, cottonseed, groundnuts, rice, wheat, spices, cottonseed, tree nuts, milk (via AFM1)	Highly hepatotoxic and carcinogenic; causes hepatocellular carcinoma (HCC), acute aflatoxicosis, immunosuppression, growth retardation, liver cirrhosis	Metabolized by CYP450 enzymes to AFB_1_‐8,9‐epoxide, forming DNA adducts (e.g., AFB_1_‐N7‐Gua), leading to mutations in tumor suppressor genes like p53; induces oxidative stress and mitochondrial dysfunction	Carpena et al. (2024), Pozarska et al. (2024), Martínez et al. (2023), Dai et al. (2022), and Benkerroum (2020b)
Aflatoxin B2 (AFB2)	*Aspergillus flavus*; *Aspergillus parasiticus*	Similar to AFB1: cereals, nuts, spices, milk products	Less potent than AFB_1_; contributes to overall aflatoxin toxicity	Similar metabolic activation as AFB_1_ but with lower efficiency; forms DNA adducts leading to mutagenesis; shares oxidative stress pathways with AFB1	Al Tamim et al. (2024), Dai et al. (2024), Khabbouchi et al. (2024), and Benkerroum (2020b)
Aflatoxin G1 (AFG1)	*Aspergillus parasiticus*; *Aspergillus nomius*	Maize, rice, oilseeds, nuts, spices, dried fruits	Hepatotoxic and carcinogenic; associated with liver damage and immunotoxicity	Similar to AFB1; forms DNA adducts and induces oxidative stress	Al Tamim et al. (2024), Dai et al. (2024), Khabbouchi et al. (2024), and Benkerroum (2020b)
Aflatoxin G2 (AFG2)	*Aspergillus parasiticus*; *Aspergillus nomius*	Same as AFG1	Least toxic among aflatoxins; contributes to cumulative exposure	Metabolized similarly to other aflatoxins but with lower potency; induces oxidative stress	Al Tamim et al. (2024), Dai et al. (2024), Khabbouchi et al. (2024), and Benkerroum (2020b)
Aflatoxin M1 (AFM1)	Metabolite of AFB1 in mammals; excreted in milk	Milk, cheese, and dairy products from animals fed AFB_1_‐contaminated feed	Carcinogenic; particularly concerning for infants consuming contaminated milk	Hydroxylated derivative of AFB1; retains DNA‐binding ability and induces oxidative stress	Pipoyan et al. (2024), Benkerroum (2020b), Djekic et al. (2020), Shehab et al. (2019), and Marchese et al. (2018)
Aflatoxin M2 (AFM2)	Metabolite from AFB2	Milk; dairy products	Potential for mutagenesis and carcinogenicity; affects growth and liver health	Similar to M1 but less potent and less studied	Okechukwu et al. (2024), Pipoyan et al. (2024), Djekic et al. (2020), and Shehab et al. (2019)

## 4 Global Occurrence and Contributing Factors of AF Contamination

### 4.1 Global Prevalence and Levels of AFs

Mycotoxigenic fungi, particularly those producing AFs, are ubiquitous across the globe and pose significant threats to food security, safety, and public health. These toxins frequently contaminate a wide range of crops and food products, especially in tropical and subtropical regions where environmental conditions favor fungal proliferation (Bonkoungou et al. 2024; Nazareth et al. 2024). AFs are secondary metabolites produced primarily by *A. flavus* and *Aspergillus parasiticus*, and their presence in agricultural commodities such as maize, groundnuts, and spices is a critical concern worldwide (Shabeer et al. 2022; Jallow et al. 2021).

Recent evidence identifies SSA as a global hotspot for AF contamination, with frequent exceedances of regulatory thresholds such as the EU limit of 10 μg/kg. Climatic conditions—high humidity, elevated temperatures, and intermittent drought—combined with substandard post‐harvest practices significantly contribute to these high contamination levels (Ngum et al. 2022). Systematic reviews and meta‐analyses have provided substantial insights into the distribution of AFs across different food matrices and geographical regions. For example, an analysis of 335 peer‐reviewed studies published between 1990 and 2023 revealed widespread AF contamination in meat, edible offal, and animal‐derived products, particularly in Southeast Asia and SSA (Nazareth et al. 2024; Jalilzadeh‐Amin et al. 2023; Pereira et al. 2019; Pleadin et al. 2021).

### 4.2 Prevalence and Levels of AFs in SSA

In SSA, AF contamination constitutes a major public health and food safety issue, driven by climatic conditions, dependence on susceptible staple crops, and inadequate post‐harvest infrastructure (Keller et al. 2021; Meijer et al. 2021). Maize, groundnuts, and sorghum—staples in the regional diet—are especially vulnerable to AF contamination, with direct implications for HCC, stunted child growth, and compromised immune function (Akullo et al. 2025; Omara et al. 2021; Chilaka and Mally 2020). Multiple studies underscore the high prevalence and intensity of AF contamination. For instance, in Tanzania, a survey of 180 groundnut and 200 maize samples found AFs in all groundnut samples and in 10%–80% of maize samples (Akullo et al. 2025; Kinyenje et al. 2023).

Similarly, a comprehensive assessment in Kenya highlighted maize and peanut contamination as major concerns, driven by environmental conditions and post‐harvest handling practices (Akullo et al. 2025; Gachara et al. 2024). Meanwhile, in eastern Ethiopia, feed samples including maize feed, total mixed rations, and wheat bran from dairy farms and local markets across Chiro, Dire Dawa, and Harar showed alarming aflatoxin prevalence (Tesfaye et al. 2024). TAFs were detected in 82.8% of samples, with a mean concentration of 54.01 ± 4.72 μg/kg. In northern Uganda, a 2023 study analyzed 363 samples of locally made complementary foods, which specifically involved a malted millet–sesame–soybean composite (MMSSC) collected across wet and dry seasons. Every sample tested positive for TAFs and aflatoxin B_1_ (AFB_1_), with concentrations ranging between 0.578–1.187 μg/kg for TAF and 0.221–0.649 μg/kg for AFB_1_ (Akullo et al. 2025; Achiro et al. 2023).

Average contamination levels were 6.37 μg/kg in groundnuts and 12.47 μg/kg in maize, with some maize samples registering as high as 162.4 μg/kg, which far surpasses international safety thresholds (Boni et al. 2021; Meijer et al. 2021). A comprehensive systematic review reported mean AFB1 levels exceeding the EU's legal limit of 5 μg/kg in the majority of maize samples, with animal feed samples containing up to 4682 μg/kg (Akullo et al. 2025). Aflatoxin contamination is particularly severe in SSA due to favorable climatic conditions, inadequate post‐harvest handling, and limited regulatory enforcement, as shown in Table 3, which synthesizes findings across multiple SSA countries.

**TABLE 3:** Summary of aflatoxin contamination in SSA countries.

Country	Aflatoxin type	Prevalence	Level (μg/kg)	Food commodity	Health problems	Economic impacts	Contributing factors	References
Tanzania	AFB_1_	Groundnuts: 92%–100%; maize: 10%–80%; > 50% of maize	Up to 150; groundnuts: mean 6.37 (max 40.31); maize: mean 12.47 (max 162.4)	Maize, groundnuts	High risk of hepatocellular carcinoma (HCC)	Trade restrictions	Climate variability, poor post‐harvest practices; low awareness among farmers	Boni et al. (2021) and Meijer et al. (2021)
Ghana	AFB_1_	35% of samples contaminated	Maize: up to 348; groundnuts: up to 348	Maize, groundnuts	Potential health risks	15% of maize and 11% of groundnut samples exceeded national limits; trade restrictions and loss of market access due to regulatory limits	Diverse Aspergillus species; lack of awareness	Asare Bediako et al. (2019) and Agbetiameh et al. (2019, 2018)
Burkina Faso	Total aflatoxins	70% contamination rate	Maize: 517; groundnuts: 277; sorghum: not specified	Maize, groundnuts, sorghum	Significant public health concern; estimated 28 liver cancer cases per 100,000 persons/year	Challenges like trade restrictions and loss of market access due to contamination	Favorable climatic conditions; inadequate storage	Akullo et al. (2025), Bonkoungou et al. (2024), and Falade et al. (2022)
Malawi	Total aflatoxins	Not specified	> 20	Maize, groundnuts	Chronic exposure risks	Reduced trade competitiveness	Pre‐harvest contamination; poor storage practices; post‐harvest handling, lack of biocontrol adoption	Akullo et al. (2025) and Njoroge (2018)
Niger	Total aflatoxins	Not specified	Maize: 659; groundnuts: 628; sorghum: 259	Maize, groundnuts, sorghum	Extremely high exposure levels; Estimated 126 liver cancer cases per 100,000 persons/year	Domestic market losses disproportionately affect smallholder farmers	Pre‐harvest contamination; inadequate storage	Falade et al. (2022)
Kenya	AFB_1_	97% contamination rate; 60% of maize	Up to 1000; 1.69–403 of Maize	Maize	High exposure leading to health risks; HCC, immunosuppression	Economic losses due to trade restriction	Warm and humid climate; poor storage conditions; permeable storage sacks	Nji et al. (2022) and Omara et al. (2021)
Nigeria	AF (unspecified); AFB1	57% contamination rate of maize; 65% of groundnuts samples	Up to 955 of maize; > 10–300 of groundnuts	Maize; groundnuts	Significant health risks; liver cancer, child stunting	Export rejections (e.g., EU standards), income loss; domestic market losses disproportionately affect smallholder farmers.	Inadequate drying and storage; lack of awareness; climate variability (droughts, humidity)	Magomya and Mbatsav (2023), Imade et al. (2021), and Michael et al. (2020)
Uganda	AFB1	High	Up to 1200	Maize and groundnuts	Potential health risks; liver cancer	Export rejections and domestic market losses	Favorable climatic conditions; poor post‐harvest handling; drought stress; inadequate storage	Akullo et al. (2023), Mwesige et al. (2023), and Sserumaga et al. (2020)
Benin	AFB1	Widespread	Dose‐dependent	Maize	Growth impairment in children	Healthcare costs	Prenatal exposure, contaminated weaning foods	Ingenbleek et al. (2019)
West Africa	Total aflatoxins	High	Up to 300	Bambara groundnuts	Liver cancer	Export rejection (EU standards)	Informal markets, poor regulatory enforcement	Ouili et al. (2025)
Southern Africa	Total aflatoxins	Lower	< 10–50	Maize, sorghum	Chronic exposure (rural areas)	Regional trade disparities	Better infrastructure but rural storage gaps	Adebo et al. (2019) and Misihairabgwi et al. (2019)

### 4.3 Prevalence and Levels of AFs in the Asians

Across Asia, AF contamination remains a significant and persistent challenge, influenced by climatic conditions, agricultural practices, and supply chain management. In China, a large‐scale study analyzing 16,604 food samples found that 34.93% tested positive for AFs. Contamination was particularly severe in peanut oil (49.14%) and corn (29.10%), with corn from Guangxi province reaching mean levels of 148.57 μg/kg—nearly 10 times the Codex Alimentarius limit (Umar et al. 2023; Chen, Liu, et al. 2022; Chen, Fang, et al. 2022). In East Asia, regulatory compliance varies widely. While Japan maintains relatively low AF levels, South Korean studies found AFB1 in 59% of herbal drug samples, often exceeding permissible limits. Taiwan has also reported AF contamination in imported peanuts and peanut‐derived products (Umar et al. 2023; Mamo et al. 2021).

In Southeast Asia, countries like Malaysia and Indonesia face ongoing challenges. In Malaysia, 45% of corn samples from the Kampong Raja region contained AFs, while 30.6% of broiler feed samples in Indonesia exceeded national safety thresholds (Khodaei et al. 2021). In the Middle East, AF M1 (AFM1) contamination in dairy products is particularly concerning. A meta‐analysis of 297,530 samples across 193 studies found contamination in 87% of dairy products, with Iran, Jordan, and Turkey reporting the highest rates. Alarmingly, pasteurized milk (99.5%), UHT milk (91.3%), and raw milk (73%) were frequently contaminated, posing serious health risks (Arghavan et al. 2025). In general, AF contamination poses a significant food safety challenge in many Asian countries, affecting a wide range of staples such as maize, rice, nuts, and spices, as illustrated in Table 4.

**TABLE 4:** Summary of AF contamination levels in various Asian countries across different food commodities.

Country	Aflatoxin type	Prevalence	Levels (ppb)	Food commodity	Health problems	Economic impacts	Contributing factors	References
India	AFB1, AFM1	High; Rice: up to 85.7% contaminated; milk: up to 99% contaminated	20–300	Groundnuts, maize, rice, milk, wheat	Historical outbreaks of aflatoxicosis; ongoing exposure risks; liver cancer, immune suppression	Loss of export markets; financial losses due to crop contamination; health care costs associated with aflatoxin‐related diseases	Monsoon climate; warm and humid climate; agricultural practices; inadequate storage; lack of awareness and control measures	Bhardwaj et al. (2023) and Umar et al. (2023)
Bangladesh	AFB1, AFB2, AFM1	Moderate; maize: 28%–37% contaminated; milk: 25% contaminated	10–150	Rice, maize, milk	Potential health risks due to consumption of contaminated food; liver damage, stunted growth in children	Economic impact due to health care costs and Reduced agricultural productivity	Poor storage conditions; lack of awareness and control measures	Jesmin et al. (2023) and Umar et al. (2023)
China	Aflatoxin B1, B2, M1	Very high; 34.9% of samples positive; corn in Guangxi: 148.57 μg/kg (8.8× CAC limit)	50–500	Corn, peanuts, peanut oil	High consumption of contaminated staples; Estimated 21,625 DALYs/year; HCC risk highest in Guangxi (0.959 cases/100,000/year); cancer, growth retardation	Significant economic losses in agriculture; Significant health burden; regional disparities in exposure and risk	High humidity; poor farming practices; regional climate variations; inadequate storage practices	He et al. (2023), Qin et al. (2023), Umar et al. (2023), Chen, Liu, et al. (2022), and Chen, Fang, et al. (2022)
Vietnam	Aflatoxin B1	Moderate	15–200	Rice, maize	Liver toxicity, potential carcinogenicity	Decreased food safety standards	Inadequate regulations	Phan et al. (2023) and Nguyen et al. (2018)
Thailand	Aflatoxin B1	Low to moderate; historical data indicates significant contamination in maize	5–100	Corn, rice	Potential health risks due to consumption of contaminated maize; allergic reactions, liver health issues	Economic losses due to reduced food export potential	Variability in climate conditions; rainy seasons leading to increased contamination; inadequate storage	Mshanga et al. (2025), Umar et al. (2023), Laut et al. (2023), and Sinphithakkul et al. (2019)
Indonesia	Aflatoxin B1	High; broiler feed: 30.6% samples exceeded allowed limits	30–400	Nuts, grains, animal feed	Potential health risks to poultry; economic losses in poultry industry; liver cancer, immune system impairment	Economic losses due to reduced poultry productivity and health care costs	Climate, storage practices; lack of awareness and control measures	Iswarawanti et al. (2024), Kristiningrum et al. (2024), and Umar et al. (2023)
Pakistan	AFB1, AFB2, AFM1	Milk: up to 99.4% contaminated; cereals: up to 95.4% contaminated		Milk, cereals (rice, maize), animal feed	High rates of stunting (40.2%), wasting (17.7%), underweight (28.9%) in children; potential link to aflatoxin exposure	Economic losses due to food wastage and health care costs; impact on child development and productivity	Hot and humid climate; limited awareness; inadequate policy framework; weak implementation mechanisms	Ajmal et al. (2022) and Naeem et al. (2022)
Nepal	AFB1	Maize: up to 42.5% contaminated		Maize	Potential health risks due to consumption of contaminated maize	Economic impact due to health care costs and reduced productivity	Inadequate storage; lack of awareness and control measures	Umar et al. (2023)
Malaysia	AFB1	Corn: 45% of samples contaminated		Corn	Potential health risks due to consumption of contaminated corn	Economic impact due to health care costs and reduced productivity	Inadequate storage; lack of awareness and control measures	Umar et al. (2023)
Iran	AFM1	Milk products exceeded ISIRI and EU standards		Milk and dairy products	Potential health risks due to consumption of contaminated dairy products	Economic impact due to health care costs and reduced productivity	Inadequate storage; lack of awareness and control measures	Arghavan et al. (2025) and Umar et al. (2023)
Yemen	AFB1	Significant contamination reported in poultry feed		Poultry feed	Losses in poultry industry due to contaminated feed	Economic losses in poultry industry	Inadequate storage; lack of awareness and control measures	Umar et al. (2023)
Saudi Arabia	AFB1	12.1% of food samples contaminated; highest in nuts and seeds		Nuts, seeds, and various food products	Estimated liver cancer risk: 0.002–0.008 cases/year/100,000 persons	Economic impact due to health care costs and reduced productivity	Improper storage practices; warm and humid climate	Arghavan et al. (2025) and Alamir et al. (2023)

### 4.4 Prevalence and Levels of AFs in Latin American

Latin America also faces widespread AF contamination, particularly in maize and peanuts—dietary staples across the region. In Costa Rica, 38.6% of maize samples and 27.8% of maize‐based products were found to contain AFs (Jallow et al. 2021). A study in western Honduras examining 631 maize samples found contamination in 109, with concentrations ranging from 1.0 to 490 μg/kg (Foerster et al. 2024). Approximately 34 samples exceeded the U.S. FDA's regulatory limit of 20 μg/kg. In Mexico, AFB1 exposure is closely linked to maize tortillas. Data from the National Health and Nutrition Survey indicate high exposure levels in regions with elevated HCC mortality, implicating AFs as a contributing factor (Monge et al. 2024). Similarly, 18% and 26% of maize product samples exceeded Mexican and EU safety limits, respectively.

Brazil has extensively documented AF contamination in peanuts (64% of samples), dairy products (AFM1 in 12.5%–48% of milk types), and cheeses such as mozzarella (100% contamination) (Foerster et al. 2024). Breakfast cereals and corn flour were also significantly affected, with AFs and other mycotoxins detected in 9% and up to 95.5% of samples, respectively. In Guatemala, maize samples exhibited AFB1 concentrations up to 2656 μg/kg, drastically surpassing safety standards (Pickova et al. 2021). Chile reported AF levels up to 176.4 μg/kg in Capsicum spices, while Brazilian rice samples showed contamination reaching 70.9 μg/kg (Pickova et al. 2021; Gilbert Sandoval et al. 2019).

### 4.5 Contributing Factors to AF Contamination

AF contamination is influenced by a complex interplay of environmental, agricultural, and socioeconomic factors. Tropical and subtropical climates with high temperatures and humidity provide optimal conditions for Aspergillus spp. to thrive and produce toxins (Bonkoungou et al. 2024; Umar et al. 2023). Improper post‐harvest practices such as inadequate drying and poor storage—further exacerbate the risk of contamination (Valencia‐Quintana et al. 2020). In Tanzania, a study found that 30.7% of raw cow milk samples contained AFM1, with 27.9% exceeding EU and local regulatory limits. Contributing factors included feeding systems, concentrate feed usage, and suboptimal storage of animal feed (Robert et al. 2023; Tadele et al. 2023).

Climate change has emerged as a significant driver of AF contamination, expanding the geographical range of Aspergillus species and intensifying contamination events in previously unaffected temperate regions. For example, increased AF levels have been reported in maize from Serbia and Croatia during abnormally hot summers (Bunny et al. 2024). Predictive models forecast that by 2040, over 89.5% of corn‐growing counties in the U.S. Corn Belt will face heightened AF risk due to rising temperatures and elevated CO_2_ levels (Kos et al. 2023).

Optimal AF production occurs at 25°C–35°C and relative humidity levels around 85%. Additional environmental factors such as low light exposure, favorable pH, and specific atmospheric gases also enhance toxin synthesis (Shabeer et al. 2022; Kumar et al. 2021). Socioeconomic disparities further compound the issue. In resource‐limited settings with weak regulatory oversight and limited public awareness, AF outbreaks are more frequent and deadly. The 2004 outbreak in Kenya, which caused 125 deaths due to contaminated maize, underscores the urgent need for robust surveillance, effective policy implementation, and educational outreach (Nazareth et al. 2024; Benkerroum 2020a; Wenndt et al. 2020; Njeru et al. 2019). In Latin America, AF contamination has been documented in a variety of staple crops and food products, with several studies reporting levels surpassing regulatory thresholds, as shown in Table 5.

**TABLE 5:** Summary of AF contamination levels in various Latin American countries across different food commodities.

Country	Aflatoxin type(s)	Prevalence & levels	Contamination levels	Food commodity	Associated health problems	Economic impacts	Contributing factors	References
Mexico	AFB1	Nixtamalized maize: 4/88 samples above detection limit (1 ng/g); estimated daily intake (EDI): 0.7–11.7 ng/kg bw/day; Rural tortillas: 18% exceeded Mexican limits; 26% exceeded EU limits; higher concentrations in fall and winter	—	Maize, tortillas	Increased cancer risk: 9–439 cases per million over 75 years; Chronic exposure linked to liver cancer; high prevalence of hepatic diseases and cirrhosis	Potential healthcare costs due to aflatoxin‐related diseases; impact on maize industry and exports	Inadequate storage practices; seasonal climate variations; high maize consumption	Gilbert Sandoval et al. (2019) and Zuki‐Orozco et al. (2018)
Guatemala	AFB1	High; maize cake: 51 μg/kg; maize: < 4 μg/kg 40%–70% (maize)	Up to 2656 μg/kg; 20–200 (maize)	Maize, maize cake, beans	High incidence of hepatocellular carcinoma (HCC); positive association between aflatoxin exposure and cirrhosis	Economic burden due to healthcare costs; potential impact on maize exports; Subsistence farming losses	Warm and humid climate; traditional maize storage methods; Rainy‐season harvest, inadequate storage	Jallow et al. (2021) and Zuki‐Orozco et al. (2018)
Costa Rica	AFB1	Maize: 50 μg/kg	—	Maize	Potential health risks due to consumption of contaminated maize	Economic impact due to health care costs and reduced productivity.	Inadequate storage; lack of awareness and control measures	Zuki‐Orozco et al. (2018)
Brazil	AFB1, AFG1, AFM1	AFB1 detected in 25%–45% of maize samples; AFG1 in 7.4%	Up to 10–120 μg/kg (maize)	Maize, peanuts, milk	Potential health risks due to consumption of contaminated maize; Liver cancer, growth retardation in children	Economic impact due to health care costs and reduced productivity; Export rejections, livestock losses	Tropical climate, Inadequate storage; lack of awareness and control measures	Jallow et al. (2021) and Gilbert Sandoval et al. (2019)
Argentina	AFB1, AFM1	15%–30% (dairy)	0.1–0.8 (milk)	Milk, maize, Peanuts	Potential health risks due to consumption of contaminated peanuts; Chronic liver disease	Economic impact due to health care costs and reduced productivity; Dairy export restrictions	Contaminated feed, humidity during harvest; Inadequate storage; lack of awareness and control measures	Jallow et al. (2021) and Gilbert Sandoval et al. (2019)
Honduras	Total AFs	17.3% of samples > 20 μg/kg	1–490 μg/kg (mean 10 μg/kg)	Maize	Liver cancer, child growth impairment	Not specified	Subsistence farming; inadequate storage	Sabillon et al. (2023)
Mexico	AFB_1_	20%–50%; 9%–18% of samples > 20 μg/kg	Up to 287.23 μg/kg; 5–80 (tortillas, chili)	Maize products (tortillas, popcorn); chili peppers	Acute hepatitis, immunosuppression; Liver cancer	Domestic market losses, trade penalties	High maize consumption; Inadequate storage; Improper drying, lack of monitoring	Zuki‐Orozco et al. (2018)
Paraguay	AFM_1_	100% in milk samples	Median 33.6 ng/kg (fluid), 1820 ng/kg (powder)	Milk, milk formulas	—	—	Contaminated feed; lack of regulation	Foerster et al. (2024)
Peru	AFB1	10%–25%	5–40 μg/kg; 20 ng/g in 6/20 samples	Capsicum, Peanuts, quinoa	Hepatotoxicity, stunting	Loss of Andean crop exports include maize, quinoa, amaranth, and various root and tuber crops	Warm, humid climate; inadequate storage; traditional drying under high humidity	Foerster et al. (2024)
Uruguay	AFB_1_, AFM_1_	AFB_1_ in 8% of sorghum; AFM_1_ in 91.8% of milk samples	—	Sorghum, milk	—	—	Contaminated feed; lack of regulation	Foerster et al. (2024)
Chile	AFT	—	Up to 176.4 μg/kg	Capsicum spices	—	—	Warm, humid climate; inadequate storage	Jallow et al. (2021)

## 5 Food Security, Health, and Economic Impact of AF Contamination

AF contamination poses substantial risks to food security, public health, and economic development, particularly in low‐ and middle‐income countries where agriculture plays a critical role in livelihoods and food systems (Gelaye 2024; Ajmal et al. 2022; Ortega‐Beltran and Bandyopadhyay 2021). AFs reduce crop yields, depress market prices, and undermine farmer income, thereby exacerbating food insecurity (Wangila 2025). In addition to agricultural losses, AF exposure is linked to severe health outcomes such as HCC, childhood stunting, and immune system suppression. The economic burden extends to healthcare expenses, loss of productivity, and diminished trade capacity (Wangila 2025; Adeyeye et al. 2021; Meijer et al. 2021). A growing body of peer‐reviewed literature underscores the multidimensional impact of AFs across food systems, public health, and economic sectors, reinforcing the urgency for integrated and sustainable mitigation strategies. Combating AF risks requires a comprehensive approach encompassing biological controls, improved pre‐ and post‐harvest practices, effective detoxification methods, and evidence‐based policies (Ajmal et al. 2022; Meijer et al. 2021).

### 5.1 Food Security Implications

AF contamination disproportionately affects staple crops such as maize, groundnuts, and cereals—essential components of diets in many African countries (Adeyeye et al. 2021). Food security, defined by reliable access to sufficient, safe, and nutritious food, is threatened on multiple fronts by AFs. These toxins compromise all four pillars of food security: availability, access, utilization, and stability (Wangila 2025; FAO et al. 2024; Clapp et al. 2022). Contamination leads to pre‐ and post‐harvest losses and diminished yields, reducing overall food availability. When contaminated crops are rejected from markets or sold at lower prices, farmers experience a decline in income, reducing their purchasing power and food access. The nutritional value of affected foods may also decline, limiting dietary diversity and contributing to malnutrition. Additionally, instability in supply chains is introduced as contaminated crops are discarded or diverted from food uses (Balan et al. 2024; Jallow et al. 2021).

In SSA, where maize and groundnuts are dietary mainstays, up to 30%–60% of crops are lost annually due to AF contamination (Jallow et al. 2021; Kumar et al. 2021; Gnonlonfin et al. 2019). These losses severely impact smallholder farmers who rely on subsistence farming. Furthermore, AFs degrade proteins and essential vitamins, diminishing the nutritional quality of consumed food and exacerbating micronutrient deficiencies (Wangila 2025; Adeyeye et al. 2021; Kumar et al. 2017). In Ethiopia, factors such as inadequate storage infrastructure, poor post‐harvest handling practices, and limited farmer awareness amplify the risk of contamination. Warm temperatures and high humidity create favorable conditions for mold proliferation, contributing to post‐harvest losses. Traditional practices—such as fermenting moldy cereals into local beverages—further entrench AF presence in the food supply (Gelaye 2024; Mamo et al. 2020).

### 5.2 Public Health Impacts

The public health implications of AF contamination are particularly severe in regions with warm and humid climates. Chronic exposure to AFs, especially AFB1, the most toxic variant—is associated with a range of health issues, including HCC, immune suppression, growth retardation in children, and reproductive complications (Balan et al. 2024; Urugo et al. 2023; Jallow et al. 2021). The synergistic effect of AFB1 and hepatitis B virus (HBV) significantly elevates the risk of liver cancer, with co‐exposed individuals facing up to 12 times higher risk of developing HCC (Ajmal et al. 2022). Globally, AF exposure is estimated to cause over 21,000 new HCC cases and approximately 19,500 deaths each year (Qin et al. 2023).

AF exposure during pregnancy and early childhood is particularly concerning. Studies from The Gambia and Kenya have shown a strong association between maternal AF exposure and adverse child health outcomes, including low birth weight, growth retardation, and increased risk of stunting and wasting (Ghantous et al. 2021; Rasheed et al. 2021; Xu, Moore, et al. 2021). These outcomes are especially prevalent in areas where staple foods are frequently contaminated. In Kenya, an acute aflatoxicosis outbreak in 2004 resulted in 125 deaths following the consumption of highly contaminated maize (Nazareth et al. 2024; Rasheed et al. 2021). These cases highlight both the chronic and acute dangers posed by AFs and the urgent need for surveillance and intervention in at‐risk regions.

### 5.3 Economics Impact

Economically, AF contamination imposes considerable costs on global food systems through reduced agricultural productivity, restricted trade, and increased public health expenditures (Gelaye 2024; Postma et al. 2023; Shabeer et al. 2022). Globally, the economic burden of AF contamination is estimated to range between $6 billion and $18 billion annually (Wangila 2025; Pal et al. 2021; Kumar et al. 2017). The financial burden is especially heavy in low‐ and middle‐income countries, where regulatory oversight and AF mitigation measures are often inadequate (Ajmal et al. 2022). SSA suffers disproportionately due to climatic conditions favorable for fungal growth and limited infrastructure for storage and testing. AF‐related losses in this region are estimated to exceed $1.2 billion annually due to export rejections, healthcare costs, and crop spoilage (Keller et al. 2021; Ingenbleek et al. 2019).

In Kenya, AF contamination in groundnuts has led to significant economic losses for smallholder farmers and the broader agricultural sector due to healthcare costs and reduced market access (Wangila 2025; Meijer et al. 2021). Nigeria has implemented cost‐effective biocontrol strategies using atoxigenic strains of *A. flavus*, which are projected to prevent 7900 to 14,200 HCC cases annually and save approximately 103,000–184,000 disability‐adjusted life years (DALYs). The cost‐effectiveness ratios (CERs) for these interventions—ranging from 5.10 to 24.8—exceed the World Health Organization's threshold for highly cost‐effective public health measures (Ajmal et al. 2022; Ricker‐Gilbert et al. 2022; Meijer et al. 2021).

In Asia, warm and humid climates continue to drive AF contamination in major crops. Countries like Indonesia, the Philippines, and Thailand report annual losses of up to $900 million due to AFs in maize and animal feed (Umar et al. 2023; Kananub et al. 2021; Sinphithakkul et al. 2019). Thailand's maize export industry has been particularly affected during rainy seasons. In China, dietary AF exposure has been linked to a loss of 21,625 DALYs annually, with significant economic impacts in provinces such as Guangxi and Guangdong due to elevated healthcare costs and lost productivity (Umar et al. 2023; Chen, Liu, et al. 2022; Chen, Fang, et al. 2022).

In Latin America, comprehensive data remain limited, but the impact of AFs is evident in major producers such as Brazil and Mexico, where maize contamination threatens both domestic food safety and international trade (Odjo et al. 2022; Chulze et al. 2021). Weak regulatory systems and insufficient monitoring exacerbate the issue, increasing the risk of contaminated products entering local and global markets (Marimón Sibaja et al. 2021; Moral et al. 2020). Strengthening legal frameworks, investing in storage infrastructure, and improving stakeholder awareness are essential to addressing these challenges.

In high‐income countries such as the United States, strict regulatory standards mitigate health risks, yet economic costs persist. The U.S. corn industry alone faces annual losses ranging from $52.1 million to $1.68 billion, especially during years with drought‐induced stress that promotes fungal proliferation (Brown et al. 2024; Yu et al. 2020; Mitchella et al. 2017). These losses are attributed to increased testing costs, price discounts for contaminated grain, and disruptions in international trade.

## 6 Sustainable and Innovative Mitigation Strategies of AF Contamination

As global food systems face increasing threats from climate change and globalization, the development of sustainable and innovative strategies to mitigate aflatoxin contamination has become essential (Karthikeyan et al. 2025; Mdindikasi et al. 2024). A holistic, multifaceted approach is required—one that integrates prevention, detection, and mitigation across the entire agricultural value chain (Ortega‐Beltran and Bandyopadhyay 2023). Effective mitigation strategies must combine biocontrol methods, environmental and technological innovations (e.g., biotechnology, nanotechnology, and predictive modeling), as well as robust educational frameworks and active community engagement (Yenew et al. 2025; Mir et al. 2021; Marshall et al. 2020). This integrated approach enables stakeholders to reduce aflatoxin contamination while fostering sustainable agricultural practices and strengthening food system resilience.

### 6.1 Sustainable and Innovative Pre‐Harvest Strategies

Introducing non‐toxic, atoxigenic strains of *A. flavus* such as those formulated into commercial products like Aflasafe or strain AF36 has consistently reduced aflatoxin levels by approximately 70%–99% in maize and groundnut field trials, using competitive exclusion to suppress toxigenic strains (Loi et al. 2020; Kagot et al. 2019; Mahato et al. 2019). When paired with GAPs, they can significantly reduce fungal colonization and aflatoxin formation in the field (Kagot et al. 2019). However, limitations include the cost and access barriers for smallholder farmers, limited awareness of health risks, potential ecological shifts in fungal populations, and uncertainties around the long‐term stability of introduced strains or unintended impacts on the Aspergillus community (Kępka‐Borkowska et al. 2025; Loi et al. 2020; Mahato et al. 2019).

The genetic strategies (e.g., gene silencing, host‐induced RNA interference) have shown promising reductions in aflatoxin in field and greenhouse studies, offer long‐term, sustainable control without chemical use, and can be integrated into broader food security programs (Kępka‐Borkowska et al. 2025; Gachara et al. 2024; Kagot et al. 2019). Their limitations include slow and resource‐intensive breeding cycles, regulatory hurdles and public resistance to GMOs, variability in efficacy across environments or genotypes, cost and stability challenges for bioactive formulations, and limited field validation beyond controlled trials (Tanveer et al. 2025; Loi et al. 2023; Ortega‐Beltran and Bandyopadhyay 2023; Joutsjoki and Korhonen 2021).

#### 6.1.1 Biocontrol With Atoxigenic Strains

Biological control technologies offer a sustainable alternative to chemical pesticides, which can harm non‐target organisms, human health, and the environment (Ivezic et al. 2025; Pandit et al. 2022; Ratto et al. 2022; Stenberg et al. 2021). One of the most effective biocontrol strategies involves the use of atoxigenic strains of *A. flavus*, which competitively exclude toxigenic strains, thereby reducing AF levels in crops such as maize, groundnuts, and sorghum (Bonkoungou et al. 2024; Mahuku et al. 2023). Among biocontrol products, Aflasafe has demonstrated the highest efficacy in reducing AF contamination (Yenew et al. 2025; Krska et al. 2022). Field applications in Mozambique using Aflasafe MWMZ01 and MZ02, each containing four native atoxigenic isolates, achieved AF reductions of 78%–98% in groundnuts and 61%–93% in maize over two years (Augusto et al. 2024; Mahuku et al. 2023). Most samples met AF limits set by the EU and US.

Additionally, these products nearly eliminated toxigenic fungi from treated soils and crops (Krska et al. 2022; Maxwell et al. 2021). In South China, native strains JS4, SI1, and SXN applied to peanut fields reduced AFs by 82.8%–87.2% while maintaining the overall fungal community structure, confirming the specificity and safety of this biocontrol method (Mamo et al. 2022). Similarly, field trials in Tanzania with Aflasafe TZ01 and TZ02 led to significant AF reductions in maize and groundnuts without disrupting Aspergillus populations (Mahuku et al. 2023; Peles et al. 2021).

Despite their promise, atoxigenic strains face significant limitations. Their efficacy is highly dependent on environmental conditions (temperature, humidity, drought stress) and agricultural practices, leading to variable results across seasons and locations (Xue et al. 2025; Dovenyi‐Nagy et al. 2020). Strain selection is critical, as atoxigenic strains must be highly competitive within the specific crop and environment; strains effective in one region may fail in another due to genetic diversity in *A. flavus* populations (Mamo et al. 2022; Chofamba 2021). Some atoxigenic isolates may still produce other metabolites like cyclopiazonic acid, requiring careful safety screening (Pekar et al. 2022; Xu, Moore, et al. 2021; Mauro et al. 2018). Moreover, novel candidate strains with promising lab or microplot performance must be validated in diverse, real‐world settings before broad adoption to ensure reliable outcomes (Ortega‐Beltran and Bandyopadhyay 2023).

#### 6.1.2 Genetic and Molecular Approaches

Genetic and molecular tools provide precise mechanisms for AF mitigation and detection (Razzaghi‐Abyaneh et al. 2022; Ortega‐Beltran and Bandyopadhyay 2021; Ojiambo et al. 2018). AF biosynthesis is governed by a gene cluster of ~30 genes in a 75‐kb region, with key regulators aflR and aflS acting as transcriptional activators (Khan et al. 2021; Caceres et al. 2020). Disruption of these genes significantly reduces toxin production (Pandey et al. 2019a, 2019b; Ojiambo et al. 2018). Advancements in genomic analyses, such as *A. flavus* pangenome studies, have identified additional candidate genes (e.g., cytochrome P450s, efflux transporters) involved in AF production (Gangurde et al. 2024; Gallo and Perrone 2021). Tools like RNA interference (RNAi) and CRISPR/Cas9 genome editing have emerged as effective methods for targeting these genes (Adegbaju et al. 2024; Stakheev et al. 2024; Králová et al. 2021). RNAi‐mediated gene silencing, targeting genes such as aflR, aflC, and aflep, has reduced AF accumulation by up to 100% in crops like peanuts using both transgenic and sprayable dsRNA approaches (Han et al. 2025; Bunny et al. 2024; Loi et al. 2023). Likewise, CRISPR/Cas9 allows for the precise knockout of AF biosynthetic genes, enabling the development of non‐aflatoxigenic strains (Adegbaju et al. 2024).

However, the deployment of crop varieties genetically resistant to *A. flavus* infection and consequent AF accumulation, represents a foundational and highly sustainable strategy within integrated AF management (IAM). Extensive research has identified and characterized AF resistant germplasm in key susceptible crops. For instance, in groundnut (peanut), lines such as 55–437, J11, PI337394F, and AR‐1 have demonstrated consistent resistance across multiple environments and studies, exhibiting mechanisms like reduced seed colonization, impaired fungal growth, and inhibition of toxin biosynthesis (Yu et al. 2024; Puppala et al. 2023; Mohammed et al. 2021; Ojiewo et al. 2020). Similarly, significant progress has been made in maize, where dedicated breeding programs have developed and utilized AF resistant inbred lines like Mp715, Mp717, Mp719, Mp420, Mp313E, TZAR101, and CML247. These lines contribute valuable quantitative trait loci (QTL) and genes conferring resistance through complex mechanisms, including physical kernel barriers (e.g., thicker pericarp, tight husk cover), enhanced kernel integrity, and biochemical defenses (Mesterhazy 2024; Ogunola et al. 2021; Smith and Williams 2021; Womack et al. 2020).

#### 6.1.3 Climate‐Smart GAPs

Climate‐adaptive practices, particularly drought‐resilient crop varieties, are vital for AF mitigation (Ortega‐Beltran and Bandyopadhyay 2021; Dovenyi‐Nagy et al. 2020). Drought conditions are known to exacerbate AF accumulation by stressing plants and promoting fungal growth (Bunny et al. 2024; Xu et al. 2022). Studies in Africa have identified maize hybrids (e.g., GH05 and GH08) that maintain low AF levels and high yields under drought and artificial fungal inoculation (Oppong et al. 2022). Other genotypes, like P31G70, showed physiological traits that conferred resistance to both drought and AF accumulation (Xu et al. 2022). Integrating these traits into breeding program enhances resilience and food safety.

#### 6.1.4 Biological Detoxification

Microbial and enzymatic detoxification offers an eco‐friendly alternative to chemical and physical AF decontamination (Liu et al. 2022; Guan et al. 2021; Pandit et al. 2022). Several Bacillus strains have shown remarkable aflatoxin B_1_ (AFB_1_) degradation capabilities. For instance, *B. velezensis* DY3108 achieved 91.5% degradation at 30°C over 96 h, while *B. amyloliquefaciens* WF2020 reduced AFB_1_ levels by over 80% within 72 h (Chen, Liu, et al. 2022; Chen, Fang, et al. 2022). Enzymes such as laccases, particularly CotA from *B. subtilis* , have emerged as efficient biocatalysts for transforming AFB_1_ into less toxic metabolites like AFQ1 (Subagia et al. 2024). Further, genetic engineering has enabled heterologous expression of AF‐degrading enzymes (e.g., arm‐adtz) in Pichia pastoris, achieving 78.9% toxin reduction within 24 h (Shu et al. 2018).

### 6.2 Sustainable and Innovative Post‐Harvest Strategies

#### 6.2.1 Advanced Storage Technologies

Post‐harvest AF prevention hinges on improved storage practices. Hermetic technologies like Purdue Improved Crop Storage (PICS) bags and metal silos offer effective protection against moisture and oxygen ingress, key factors in fungal proliferation (Ngoma et al. 2024; Kaburi et al. 2023; Udomkun et al. 2017). In Zimbabwe, a randomized controlled trial found significantly lower AFB_1_ levels in maize stored in PICS bags compared to traditional methods. Additionally, hermetic storage improved grain quality and market value, enhancing farmers' income (Kaburi et al. 2023; Dijkink et al. 2022).

#### 6.2.2 Cold Plasma (CP) Technology

Cold plasma (CP), a novel, eco‐friendly, nonthermal food processing technology, has emerged as a powerful tool for the detoxification of AFs in various food commodities (Javed et al. 2025; Liu et al. 2024). Generated by applying electrical energy to gases such as air, argon, or helium, CP produces reactive oxygen and nitrogen species (RONS), UV photons, and charged particles that break down aflatoxin molecules without significantly affecting the nutritional and sensory quality of food (Molina‐Hernandez et al. 2025; Urugo et al. 2023; Hoppanová and Kryštofová 2022). Studies have demonstrated the high efficacy of CP in degrading AFB_1_ in diverse substrates.

For example, Liu et al. (2024) reported over 90% reduction in AFB_1_ in maize kernels after 10 min of CP exposure. Similarly, Javed et al. (2025) achieved 95% degradation in dried figs using dielectric barrier discharge (DBD) CP treatment. The technology's key advantages lie in its nonthermal nature, which allows the preservation of heat‐sensitive nutrients, its low chemical residue, which enhances food safety, and its rapid treatment times, making it suitable for potential scalability in industrial applications. Furthermore, CP is being integrated into packaging materials to actively prevent aflatoxin formation during storage and transportation (Sharma et al. 2025; Karthik et al. 2024). Despite these promising attributes, practical challenges such as high equipment costs, limited optimization for large‐scale deployment, and the need for regulatory approvals remain barriers to widespread adoption.

#### 6.2.3 Optical Sorting and Digital Technologies

Optical and hyperspectral imaging technologies are increasingly applied for the post‐harvest detection and removal of AF‐contaminated kernels. These systems work by identifying fungal‐infected or discolored grains based on visual or spectral differences, thus enabling the effective sorting out of contaminated products and significantly reducing aflatoxin levels in the final food supply (He et al. 2021; Aoun et al. 2020). Complementing these technologies, digital innovations such as artificial intelligence (AI)‐based AF prediction models and blockchain systems for traceability are enhancing the monitoring and mitigation of AFs across the value chain (Gbashi and Njobeh 2024; Krska et al. 2022). Furthermore, the availability of portable, smartphone‐linked sensors and fluorescence‐based field detection devices is making real‐time, low‐cost aflatoxin screening accessible to farmers, traders, and processors in low‐resource settings.

#### 6.2.4 Community‐Based and Farmer‐Led Innovations

Engaging smallholder farmers through practical training, early warning systems, and participatory innovation has proven effective in improving post‐harvest practices and reducing AF exposure. In countries such as Ethiopia, Nigeria, and Kenya, community‐based interventions like shared drying centers, improved harvesting protocols, and awareness campaigns have led to measurable reductions in contamination (Yenew et al. 2025; Maxwell et al. 2021). Importantly, farmer cooperatives and women‐led initiatives have been instrumental in promoting and implementing low‐cost solutions such as solar dryers, grain moisture meters, and hermetic storage bags. These grassroots efforts not only enhance food safety but also contribute to building local resilience and empowering vulnerable communities through increased knowledge and economic participation.

### 6.3 Natural Inhibitors and Nanotechnology

As synthetic chemical treatments raise concerns over toxicity and environmental safety, attention has increasingly shifted toward more sustainable and innovative mitigation strategies. Among these, natural inhibitors and nanotechnology‐based interventions have garnered significant interest due to their eco‐friendly nature and effectiveness in preventing aflatoxin contamination (Kibugu et al. 2024; Chadha et al. 2022; Hoque et al. 2021; Thiruvengadam et al. 2018).

#### 6.3.1 Natural Inhibitors

Natural inhibitors are derived from plant extracts, microbial metabolites, or food‐grade compounds and work primarily by inhibiting the growth of aflatoxigenic fungi or suppressing aflatoxin biosynthesis pathways (Ahmad et al. 2022). A wide range of plant‐derived substances, including essential oils and phytochemicals, have demonstrated potent antifungal and anti‐aflatoxigenic properties. Essential oils extracted from clove, thyme, cinnamon, and oregano contain bioactive compounds such as eugenol, thymol, and cinnamaldehyde, which disrupt fungal cell membrane integrity and inhibit toxin production. These oils have shown significant efficacy in reducing AFB_1_ production (Linan‐Atero et al. 2024; Aguilar‐Veloz et al. 2020). Additionally, phenolic compounds including flavonoids, tannins, and phenolic acids from spices like turmeric and garlic have been found to modulate fungal metabolism and suppress AF gene expression. Herbal extracts from ginger and moringa have also been reported to reduce AF levels in stored grains, further supporting the role of botanical compounds in post‐harvest management (Medalcho et al. 2023; Victor Jeyaraj et al. 2022; Mubeen et al. 2020).

Bentonite, a naturally occurring adsorbent clay composed primarily of montmorillonite, has been extensively studied for its role in mitigating aflatoxin contamination due to its high surface area, swelling capacity, and strong adsorption properties, which collectively enhance toxin binding while minimizing nutrient loss (Kihal et al. 2023; Wang et al. 2019). Recent research confirms its high efficacy, often exceeding 90% reduction in aflatoxin B1 (AFB1) in the gastrointestinal tract, thus reducing its bioavailability and toxic effects in vitro and in vivo (José Mendes dos Reis et al. 2024; Hamad et al. 2023; Alharthi et al. 2022). Studies conducted over the past five years confirm that dietary inclusion of bentonite in poultry, cattle, and swine feed significantly reduces aflatoxin‐induced liver damage, improves feed conversion rates, and lowers aflatoxin residues in milk and meat (Kolawole et al. 2022; Popescu et al. 2022; Ghazalah et al. 2021). Overall, adsorbent clays represent a cost‐effective and scalable solution to aflatoxin mitigation, especially when deployed alongside good agricultural and storage practices.

#### 6.3.2 Nanotechnology

Nanotechnology presents a frontier in AF mitigation through its ability to enhance detection, adsorption, and neutralization of toxins with unprecedented precision (Bajpai et al. 2018; Thiruvengadam et al. 2018). Nanomaterials such as gold nanoparticles (AuNPs), carbon nanotubes, and quantum dots have enabled the development of nano‐biosensors for highly sensitive and real‐time AF detection (Guruprasath et al. 2024; Shafiq et al. 2020). Moreover, nano‐adsorbents—engineered from materials like nano‐clays, nanosilica, and activated carbon—are increasingly used to bind and sequester AFs in food and animal feed. Silver nanoparticles (AgNPs), known for their broad‐spectrum antimicrobial activity, have been shown to inhibit both fungal growth and aflatoxin biosynthesis (Chadha et al. 2022).

Recent advancements include the use of chitosan‐based biodegradable nanoparticles loaded with antifungal agents, which significantly enhance the bioavailability, stability, and efficacy of natural inhibitors (Detsi et al. 2020). Encapsulation techniques, such as embedding essential oils or phytochemicals within nano‐carriers, further improve their delivery and controlled release, increasing their antifungal effectiveness in food systems. Notably, nano‐encapsulated cinnamon oil has demonstrated prolonged antifungal activity, contributing to extended shelf life and enhanced safety of stored grains (Linan‐Atero et al. 2024).

### 6.4 Integrated and Emerging Approaches

As AF contamination remains a multifaceted challenge, integrated and technology‐driven approaches are gaining prominence in global mitigation efforts. Emerging innovations, including the IoT, blockchain technology, predictive modeling, and policy‐based interventions, offer promising avenues for transforming how food systems prevent, detect, and respond to aflatoxin risks.

#### 6.4.1 Internet of Things (IoT)

The application of IoT technologies in agricultural and post‐harvest systems provides real‐time monitoring of environmental parameters critical to AF development, such as temperature and humidity. In Indonesia, Hadipernata et al. (2021) demonstrated the effectiveness of IoT‐enabled storage systems in maintaining maize moisture content below 14%, thereby suppressing fungal growth and AF production. These systems utilized sensors and actuators for automated environmental control, showcasing the potential of IoT to ensure optimal storage conditions. When integrated with AI, IoT enhances predictive capabilities, enabling early detection and mitigation of aflatoxin risks. As Mu et al. (2024) emphasized, the fusion of AI, big data analytics, and IoT within food safety early warning systems provides critical insights that facilitate timely and proactive interventions.

#### 6.4.2 Blockchain Technology for Traceability

Blockchain technology, with its decentralized and tamper‐proof ledger system, is revolutionizing traceability and accountability within food supply chains. By recording each transaction along the value chain, blockchain enables swift identification and resolution of contamination sources (Duan et al. 2024; Zarba et al. 2024). This transparency enhances consumer trust and improves regulatory oversight. Singh et al. (2023) reported that blockchain integration could reduce the time required to trace contaminated food products from several days to just seconds. The synergy between blockchain and IoT further strengthens data integrity and environmental monitoring. IoT sensors collect data on storage and transport conditions such as temperature and humidity, which are then securely logged in blockchain systems, allowing for data‐driven decision‐making to prevent aflatoxin outbreaks (Sri Vigna Hema and Manickavasagan 2024).

#### 6.4.3 Predictive Modeling

Predictive modeling is a crucial tool for risk assessment and early intervention in AF management. Leveraging statistical, mechanistic, and machine learning approaches, these models help stakeholders anticipate contamination risks and implement preventive measures. For example, Branstad‐Spates et al. (2023) developed a gradient boosting machine (GBM) model using meteorological data, soil properties, and historical contamination records to predict AF levels in corn in Iowa, achieving high predictive accuracy. Similarly, in Texas, Castano‐Duque et al. (2025) combined satellite‐based geospatial data with environmental variables to model AF outbreaks in maize. These approaches underscore the potential of integrating remote sensing and big data for spatially and temporally precise risk prediction.

#### 6.4.4 Policy and Regional Initiatives

AF control also necessitates strong governance frameworks and transboundary collaboration. The African Union's Strategic Framework for Holistic AF Control exemplifies efforts to harmonize regulations, raise awareness, and strengthen institutional capacity across member states (African Union 2023). The framework's recent expansion to include 12 additional countries, such as Ethiopia, Ghana, and Kenya, signals a growing continental commitment to combat AFs. Moreover, regional bodies such as the Comité Sahélien des Pesticides (CSP), operating under the Permanent Interstate Committee for Drought Control in the Sahel (CILSS), facilitate the joint registration of biocontrol agents like Aflasafe, enabling cost‐effective, cross‐border deployment (Bonkoungou et al. 2024; Ortega‐Beltran and Bandyopadhyay 2023). These coordinated initiatives highlight the importance of political will, regional harmonization, and public‐private partnerships in developing sustainable AF mitigation strategies.

Recent studies underscore the effectiveness of biocontrol agents made from native, non‐aflatoxigenic strains of *A. flavus*, notably the “*Aflasafe*” products developed by the International Institute of Tropical Agriculture (IITA) and partners. Field trials across Burkina Faso, Mali, Niger, and Togo demonstrated aflatoxin reductions of 57%–100% in treated maize, groundnut, and sorghum even after prolonged storage under suboptimal conditions (Yenew et al. 2025; Bonkoungou et al. 2024). In Kenya, randomized field interventions showed that basic training in the application of biocontrol reduced aflatoxin in maize by ~34%, while additional timely reminders boosted reduction levels to ~52% (Chilaka et al. 2022; Mutegi et al. 2018). Malawi leverages satellite data and weather forecasting to predict high‐risk zones, directing government‐subsidized biocontrol applications to vulnerable smallholders before contamination occurs (Warnatzsch et al. 2020).

Meta‐analyses and systematic reviews further support combining technological tools like Aflasafe with One Health approaches, which integrate agricultural practice, environmental management, public health education, and community awareness to yield sustainable reductions in contamination levels of up to ~90% (Yenew et al. 2025; Ortega‐Beltran and Bandyopadhyay 2021). A comprehensive overview of these sustainable and emerging approaches ranging from natural inhibitors and nanotechnology to digital innovations and policy frameworks is presented in Table 6, highlighting their mechanisms, advantages, and applicability across various points of the food value chain.

**TABLE 6:** Summary of sustainable and innovative mitigation strategies for aflatoxin contamination in global food systems.

Strategies	Principles	Advantages	Limitations	Applications	Efficacy in food matrices	References
Biocontrol with atoxigenic strains	Utilizes non‐toxigenic *Aspergillus flavus* strains to competitively exclude toxigenic strains in crops	Eco‐friendly, cost‐effective, long‐term efficacy; significant aflatoxin reduction (up to 99%)	Requires region‐specific strain selection; efficacy influenced by environmental conditions, regulatory hurdles	Applied in maize, groundnuts, and peanuts across Africa, Italy, and China	Demonstrated 76%–100% reduction in aflatoxins in maize and groundnuts in Ghana; over 90% reductions in maize in Italy	Luis et al. (2022), Mamo et al. (2022), Agbetiameh et al. (2020), and Mauro et al. (2018)
Genetic and molecular approaches	CRISPR, RNAi, or gene editing to disrupt aflatoxin biosynthesis pathways	Potential for long‐term resistance; can be integrated into existing breeding programs, high specificity, reduced chemical use	Regulatory hurdles; public acceptance issues; potential off‐target effects; technical complexity	Development of transgenic maize and groundnut varieties with enhanced resistance	Showed 60%–80% reduction in grains; experimental success in lab models	Jallow et al. (2021), Caceres et al. (2020), and Ojiambo et al. (2018)
Hermetic storage	Creates airtight storage conditions to inhibit fungal growth and aflatoxin production post‐harvest through oxygen deprivation	Simple technology; scalable; preserves grain quality; reduces post‐harvest losses	Initial cost of hermetic bags; requires proper usage and maintenance; less effective for already contaminated crops	Used for storing maize, groundnuts, rice, and other grains in various regions	90%–95% reduction in stored grains; limited efficacy in high‐moisture conditions	Ngoma et al. (2024), Kaburi et al. (2023), and Udomkun et al. (2017)
Nanotechnology	Applies nanoparticles or nano‐adsorbents (e.g., nanozymes, magnetic nanoparticles, clay, and chitosan) for aflatoxin detection and degradation	High sensitivity and specificity; potential for real‐time detection and detoxification, versatile application	Cost, limited field application data; concerns about nanoparticle safety and regulatory approval	Development of nano‐sensors for aflatoxin detection in food products; experimental detoxification methods, additive treatments	Up to 96% aflatoxin removal in vegetable oils using nanozymes; efficacy in solid matrices under investigation; variable efficacy in dairy	Loi et al. (2023), Thirugnanasambandan and Gopinath (2023), Detsi et al. (2020), and Shafiq et al. (2020)
Biological detoxification	Utilizes microorganisms (e.g., *Flavobacterium*) or enzymes (e.g., laccase) to degrade aflatoxins into less toxic compounds	Eco‐friendly; specific; potential for integration into food processing; retains nutritional quality	Slow kinetics; requires optimization; incomplete degradation; possible formation of unknown metabolites; scalability challenges	Application in feed treatment and food processing industries.	Demonstrated aflatoxin reduction in laboratory settings; 50%–90% degradation in liquid matrices; lower efficacy in solid foods.	Subagia et al. (2024), Loi et al. (2023), Chen, Liu, et al. (2022), Chen, Fang, et al. (2022), and Guan et al. (2021)
Blockchain technology	Implements decentralized digital ledgers to enhance traceability and transparency in the food supply chain contaminations	Improves supply chain accountability; facilitates rapid response to contamination events; reduces fraud; real‐time monitoring	Requires high technological infrastructure; adoption barriers in low‐resource settings; data privacy concerns	Supply chain management (grains, spices); Pilot projects in tracking grain quality and safety from farm to market.	Potential to reduce aflatoxin exposure by ensuring contaminated batches are identified and removed; improves recall accuracy but does not reduce contamination	Bonkoungou et al. (2024), Duan et al. (2024), Zarba et al. (2024), and Singh et al. (2023)
Predictive modeling	AI/ML models predict contamination risks using agronomic, climate and environmental data to forecast aflatoxin risk and inform mitigation strategies	Enables proactive mitigation; cost‐effective; supports decision‐making for interventions	Model accuracy depends on data quality; may require continuous updates	Development of early warning systems for aflatoxin outbreaks in agriculture and food systems	75%–90% accuracy in maize/peanuts; effectiveness varies; models have successfully predicted high‐risk periods in certain regions	Castano‐Duque et al. (2025), Inglis et al. (2024), Branstad‐Spates et al. (2023), and Keller et al. (2021)
Purdue improved crop storage (PICS) bags	Hermetic triple‐layered bags create an oxygen‐deprived environment, inhibiting mold and insect activity	Chemical‐free, reusable, cost‐effective, suitable for smallholders, maintains grain quality, Reduces post‐harvest losses	Requires proper sealing and thorough drying before storage; susceptible to rodent damage; potential need for new storage facilities	Post‐harvest storage of grains like maize, cowpeas, sorghum, peanuts, and grains in sub‐Saharan Africa and Asia	Effective in reducing aflatoxin levels in stored grains (e.g., maize: 90%–95% reduction); maintains quality over several months	Jyothi et al. (2024), Momanyi et al. (2022), Mompremier et al. (2022), Rabe et al. (2021), and Baributsa and Njoroge (2020)
Internet of Things (IoT)	Utilizes sensors and connectivity to monitor and control environmental conditions affecting aflatoxin production	Real‐time monitoring, predictive analytics, data‐driven decision‐making, scalable	High initial setup cost; requires technical expertise; connectivity issues in remote areas	Monitoring storage conditions, grain silos, warehouses, supply chain management in industrialized regions, predictive maintenance	Enhances early detection and prevention strategies; limited efficacy in smallholder settings, effectiveness depends on integration and user response	Mu et al. (2024), Ataei Kachouei et al. (2023), and Hadipernata et al. (2021)
Cold plasma technology	Nonthermal plasma generates reactive species (e.g., ozone) that degrade aflatoxins and inactivate fungi	Chemical‐free, low‐temperature, minimal impact on food quality, rapid processing	High energy use; limited penetration in dense food matrices; costly setup; scalability challenges; regulatory approvals pending	Decontamination of cereals, nuts, spices, and dried fruits; surface sterilization; and processed foods in high‐value supply chains	Up to 99% reduction in aflatoxins in various matrices; effectiveness varies with food type and treatment parameters	Mahmoud et al. (2025), de Oliveira et al. (2025), and Misra et al. (2018)
Natural inhibitors	Use of natural compounds (e.g., essential oils, plant extracts, microbial metabolites) to inhibit aflatoxin‐producing fungi	Eco‐friendly, potential health benefits, consumer acceptance, multi‐functional (antimicrobial)	Variability in efficacy; potential sensory impacts; regulatory hurdles for food applications	Pre‐ and post‐harvest treatment of grains, nuts, and spices; integration into packaging materials	Significant reduction in aflatoxin levels; moderate in cereals (e.g., 50%–80% inhibition); low in high‐moisture foods (e.g., dairy); effectiveness depends on concentration and application method	Ahmad et al. (2022) and Adebo et al. (2021)

## 7 Novel Analytical Techniques for AF Detection in Agri‐Food Systems

To monitor and enforce regulatory limits, fast, accurate, and reliable means of detecting and quantifying AFs in foodstuffs are required. Detection methods need to be sensitive, accurate, reproducible, and easy to use. Aflatoxin detection procedures are multistage. They involve sampling, extraction, purification, enrichment, analysis, and post‐analysis data interpretation. Traditional methods (chromatographic, immunochemical, and spectroscopic) face limitations in speed, cost, portability, and multiplexing (Balan et al. 2024; Jallow et al. 2021). Recent advancements in analytical techniques for aflatoxin estimation in agri‐food systems have introduced highly sensitive, rapid, on‐site, and cost‐effective alternatives to traditional methods (Alameri et al. 2023; Okechukwu et al. 2024). Several methods are usually employed for the quantification of AFs in food commodities.

### 7.1 Advanced Sample Extraction and Chromatographic Techniques

Molecularly imprinted polymer (MIP)‐based solid phase extraction, combined with high‐performance liquid chromatography (HPLC‐FLD), has demonstrated excellent selectivity and recovery for multiple AFs (AFB_1_, AFB_2_, AFG_1_, AFG_2_), achieving limits of detection ranging from 0.06 to 0.21 μg/kg, quantification from 0.20 to 0.69 μg/kg; recovery rates 79%–109% with precision < 5%, fulfilling EU regulatory standards, in complex food matrices such as maize and peanuts (Mbisana et al. 2025; Tittlemier et al. 2025; Wood et al. 2023; Schincaglia et al. 2023; Singh and Mehta 2020).

### 7.2 Miniaturized & Microfluidic Platforms

Microfluidic platforms coupled with nano‐LC–MS/MS are further pushing sensitivity boundaries, detecting AFs at parts‐per‐trillion levels with minimal sample volume and rapid throughput (Tittlemier et al. 2025). Researchers used a nano‐LC microfluidic chip with triple‐quadrupole MS for AFs, including AFB_1–2_, AFG_1–2_, AFM_1_, in peanut products. Achieved LODs are 0.004–0.008 μg/kg and the linear quantitation range is down to 0.048 μg/kg, with 10× enhanced sensitivity over conventional HPLC–MS/MS (Schincaglia et al. 2023; Man et al. 2017).

### 7.3 Biosensors, Immunoassays, and Aptamer‐Based Platforms

Aptamer‐based biosensors, particularly those integrated with fluorescence resonance energy transfer (FRET) systems, have emerged as highly promising tools for aflatoxin estimation in agri‐food systems due to their exceptional sensitivity, specificity, and potential for on‐site application (Victor Jeyaraj et al. 2022; Li et al. 2021). These biosensors utilize single‐stranded DNA or RNA aptamers that bind selectively to AFs, enabling rapid detection even in complex food matrices. Recent studies have demonstrated the use of graphene oxide and gold nanoclusters in FRET‐based aptasensors to detect aflatoxin B1 (AFB_1_) in maize, achieving detection limits in the picomolar range (as low as 6.7 pg/mL) (Li et al. 2021; Guo et al. 2020). Another notable approach employed quantum dot‐labeled aptamers and magnetic separation, allowing for dual‐target detection of AFB_1_ and fumonisin B1 in corn with minimal matrix interference (Li et al. 2024, 2021).

### 7.4 Spectroscopy–Machine Learning‐Based Approaches

Fluorescence spectroscopy combined with machine learning has gained traction as a rapid, non‐destructive method for aflatoxin estimation in agri‐food systems, offering an efficient alternative to conventional chromatographic techniques (Xu et al. 2024). This approach captures the unique fluorescence signatures emitted by contaminated samples when exposed to specific wavelengths of light, enabling the differentiation of aflatoxin presence and concentration. A recent study by Bertani et al. (2020) demonstrated the effectiveness of this method on ground almond samples, where fluorescence spectral data were processed using support vector machine (SVM) algorithms to classify contamination levels. The model achieved an accuracy of 94% with a detection threshold of approximately 6.4 ng/g, aligning with regulatory limits for aflatoxin B1.

### 7.5 Emerging Imaging Methods

Although not aflatoxin–specific yet, dual‐mode reflectance/transmittance multispectral imaging combined with AI algorithms has successfully identified adulteration in food matrices with > 95% accuracy and up to 99% in merged mode, indicating potential for adaptation to aflatoxin detection in grains and oils (Kabir et al. 2025; Lun et al. 2025; Udayanga et al. 2023; Soni et al. 2022). Additionally, spectroscopic and imaging techniques such as optical fiber‐coupled sensors and multispectral imaging powered by AI enable non‐destructive, real‐time aflatoxin screening of whole grain or nut batches (Jia et al. 2024; Raki et al. 2024; Bertani et al. 2020). These novel methods together mark a shift toward decentralized, high‐throughput aflatoxin monitoring, essential for safeguarding food safety across diverse agri‐food systems.

## 8 Challenges and Future Directions

AF contamination, primarily caused by Aspergillus species, continues to pose a significant threat to global agri‐food systems, particularly in tropical and subtropical regions due to its severe implications for public health, food security, and economic development (Bhardwaj et al. 2023; Jallow et al. 2021). Recent studies have emphasized the role of climate change as a major driver of increased AF prevalence. Rising global temperatures, shifting precipitation patterns, and higher humidity levels create favorable conditions for fungal growth in staple crops such as maize, groundnuts, and other cereals. These fungi not only thrive under changing climatic conditions but also produce AFs that are highly stable and resistant to conventional food processing methods (Bunny et al. 2024; Umar et al. 2023; Urugo et al. 2023; Nji et al. 2022).

Although various physical and chemical mitigation strategies have been explored, the use of chemical preservatives raises significant health and environmental concerns, limiting their widespread acceptance (Gachara et al. 2024; Ortega‐Beltran and Bandyopadhyay 2023; Kumar et al. 2022). Socioeconomic disparities further compound these challenges, particularly for smallholder farmers in low‐income countries who often lack access to appropriate technologies, infrastructure, and training. These constraints perpetuate cycles of contamination, food insecurity, and trade restrictions. Thus, the development of climate‐resilient agricultural practices and equitable dissemination of mitigation technologies is essential for effective long‐term control (Balan et al. 2024; Nazareth et al. 2024).

The detection and monitoring of AFs also remain critical challenges. Conventional methods such as HPLC and enzyme‐linked immunosorbent assays (ELISA) are accurate but often expensive, time‐consuming, and reliant on well‐equipped laboratories, rendering them impractical for routine use in low‐resource settings (Kumar et al. 2022). In response, there has been growing interest in sustainable and innovative approaches, including the development of resistant crop varieties, biotechnology applications, advanced food processing methods, nanotechnology, and machine learning‐based predictive models. These tools offer promising solutions for real‐time, on‐site detection and targeted intervention. However, as Nazareth et al. (2024) highlight, these innovations require rigorous field validation across diverse agro‐ecological zones and capacity building among stakeholders to ensure successful adoption. The disconnect between technological innovation and on‐the‐ground implementation remains a critical barrier to effective AF risk reduction (Jallow et al. 2021).

In addition, limited awareness among producers and weak regulatory enforcement further exacerbate the issue. Studies indicate that more than 70% of farmers are unaware of AF risks and management strategies, pointing to a pressing need for comprehensive training and education initiatives. The absence or poor enforcement of food safety regulations in many low‐ and middle‐income countries undermines control efforts and allows contaminated products to circulate within and beyond national markets (Balan et al. 2024; Ortega‐Beltran and Bandyopadhyay 2023). Looking forward, future strategies must prioritize stronger policy alignment, regulatory harmonization, and cross‐sectoral collaboration. The current lack of standardized AF limits across countries creates barriers to international trade and diminishes incentives for compliance, especially in regions where enforcement mechanisms are weak. Initiatives such as the Partnership for Aflatoxin Control in Africa (PACA) and the Codex Alimentarius guidelines developed by the FAO and WHO offer valuable frameworks, but their effectiveness depends on sustained political will and financial investment.

Incorporating AF control measures into broader climate adaptation and food security policies can further strengthen mitigation efforts. Digital platforms that facilitate knowledge exchange, remote training, and real‐time surveillance can enhance early warning systems and improve coordination across stakeholders. Notably, adopting a One Health approach, which integrates agricultural, environmental, and public health dimensions, has been proposed as a holistic strategy to address AF risks while promoting sustainable food systems (Yenew et al. 2025). Investment in advanced detection technologies and the expansion of regional and national surveillance programs will be vital to proactively manage contamination events and protect public health (Yenew et al. 2025; Bunny et al. 2024; Nazareth et al. 2024; Jallow et al. 2021).

## 9 Conclusions

AF contamination continues to pose a significant threat to global food systems, particularly in tropical and subtropical regions such as SSA and Asia, where environmental conditions, limited infrastructure, and socioeconomic disparities heighten the risk. This comprehensive review has synthesized the current state of knowledge regarding the prevalence, health implications, contributing factors, and mitigation strategies for AF contamination. Key insights highlight the influence of climate change as a central driver of contamination, intensifying the proliferation of Aspergillus species through elevated temperatures and erratic rainfall patterns. Biocontrol strategies, particularly the use of atoxigenic strains, have shown notable success in reducing AF levels by up to 100% under certain conditions. Additionally, innovative detoxification technologies including CP treatment, nanotechnology applications, and ozone‐based decontamination offer promising alternatives to conventional chemical and physical methods. However, their adoption remains limited due to cost, technical complexity, and lack of localized validation.

Despite scientific and technological progress, several critical gaps persist. These include limited access to accurate and affordable detection methods, insufficient awareness and training among farmers, and fragmented regulatory frameworks across regions. Socioeconomic inequities further constrain the capacity of smallholder farmers to implement preventive strategies, reinforcing cycles of contamination and food insecurity. To overcome these multifaceted challenges, integrated and forward‐looking solutions are essential. Strengthening climate‐smart GAPs, harmonizing food safety standards, and expanding education and outreach programs are fundamental steps toward building aflatoxin‐resilient systems. Emerging tools such as the IoT, blockchain for traceability, and machine learning‐based predictive analytics can transform surveillance and early warning capabilities, especially when embedded within a coordinated data‐driven framework. Importantly, a One Health approach linking agricultural, environmental, and public health sectors should be mainstreamed into AF control strategies to ensure holistic risk reduction and sustainable food system transformation. Moving forward, targeted investments in research, cross‐sectoral collaborations, and equitable dissemination of technologies will be critical to safeguarding food quality and protecting vulnerable populations from the enduring burden of AF exposure.

We strongly urge prioritization of protection for children under 15 and individuals with chronic illnesses against aflatoxin exposure, given their heightened vulnerability to severe health impacts. To mitigate risks, local authorities must proactively leverage existing extension networks for community‐wide education on safe crop storage, cleaning, and handling practices throughout the food supply chains. To proactively contain potential outbreaks of acute aflatoxicosis, we mandate coordinated action by Sectoral Ministries (Agriculture, Trade, Health, and Education) and key stakeholders to immediately establish a nationwide, real‐time surveillance system tracking acute jaundice incidence in humans. This system must serve as an early warning mechanism, triggering rapid response protocols. Concurrently, we demand the implementation and rigorous enforcement of legally binding limits for aflatoxin contamination across all food value chains, with zero tolerance for non‐compliance.

Finally, we recommend that breeding strategies prioritize the development of crop lines with both direct and indirect resistance to aflatoxin contamination. Direct resistance includes physical or biochemical traits that actively suppress fungal growth or toxin production, such as tightly closed maize husks and robust ear structures. Indirect resistance refers to traits that reduce susceptibility to infection, including early maturity, strong husk coverage, and drought tolerance. Deploying resistant varieties not only lowers aflatoxin levels and enhances food safety but also offers a cost‐effective, sustainable solution for long‐term crop resilience.

## Author Contributions

**Eyasu Yohannis:** conceptualization (lead), data curation (lead), formal analysis (lead), investigation (lead), methodology (lead), visualization (lead), writing – original draft (lead). **Markos Makiso Urugo:** data curation (equal), investigation (equal), methodology (equal), supervision (equal), validation (equal), visualization (equal), writing – review and editing (equal). **Tilahun A. Teka:** data curation (equal), investigation (equal), methodology (equal), supervision (equal), validation (equal), visualization (equal), writing – review and editing (equal). **Paulos Getachew:** methodology (equal), supervision (equal), validation (equal), writing – review and editing (equal). **Yetenayet B. Tola:** methodology (equal), supervision (equal), validation (equal), writing – review and editing (equal). **Sirawdink Fikreyesus Forsido:** methodology (equal), supervision (equal), validation (equal), writing – review and editing (equal). **Yikeber Simachew Kebede:** data curation (equal), methodology (equal), validation (equal), writing – review and editing (equal). **Tadesse Fikre Teferra:** methodology (equal), supervision (equal), validation (equal), visualization (equal), writing – review and editing (equal).

## Conflicts of Interest

The authors declare no conflicts of interest.

## Data Availability

The authors have nothing to report.
